# Analog Circuit Fault Diagnosis via Joint Cross-Wavelet Singular Entropy and Parametric t-SNE

**DOI:** 10.3390/e20080604

**Published:** 2018-08-14

**Authors:** Wei He, Yigang He, Bing Li, Chaolong Zhang

**Affiliations:** 1 School of Electrical Engineering and Automation, Hefei University of Technology, Hefei 230009, China; 2 School of Electrical Engineering, Wuhan University, Wuhan 430072, China; 3 School of Physics and Electronic Engineering, Anqing Normal University, Anqing 246011, China

**Keywords:** analog circuit, fault diagnosis, cross wavelet transform, Tsallis entropy, parametric t-distributed stochastic neighbor embedding, support vector machine

## Abstract

In this paper, a novel method with cross-wavelet singular entropy (XWSE)-based feature extractor and support vector machine (SVM) is proposed for analog circuit fault diagnosis. Primarily, cross-wavelet transform (XWT), which possesses a good capability to restrain the environment noise, is applied to transform the fault signal into time-frequency spectra (TFS). Then, a simple segmentation method is utilized to decompose the TFS into several blocks. We employ the singular value decomposition (SVD) to analysis the blocks, then Tsallis entropy of each block is obtained to construct the original features. Subsequently, the features are imported into parametric t-distributed stochastic neighbor embedding (t-SNE) for dimension reduction to yield the discriminative and concise fault characteristics. Finally, the fault characteristics are entered into SVM classifier to locate circuits’ defects that the free parameters of SVM are determined by quantum-behaved particle swarm optimization (QPSO). Simulation results show the proposed approach is with superior diagnostic performance than other existing methods.

## 1. Introduction

With the fast development of electronic science and technology, fault diagnosis and testing as fundamental tasks in preventive maintenance of electronic systems play a vital role in reliability of the product and promoting industrial development [1,2]. It is estimated that testing covers one third of the cost of the product, and majority of the testing is due the testing of the analog parts of the mixed signal circuits [3,4]. Due to continuous parameter and tolerance of analog components, and lack of test nodes, the diagnostics approaches of analog circuits are far less advanced, comparing with well-developed automatic fault diagnosis methodologies for digital circuits. Consequently, there is a pressing need to explore effective fault diagnosis and testing approaches to prevent fault enlargement and guarantee analog electronic system reliable operation.

Faults in analog circuits can be categorized into soft faults and hard faults. Soft faults result in system performance degradation where the parameters of components only deviate from the normal values exceeding the tolerance range. The causes for soft faults mainly include: the aging of an electronic system, fabrication tolerance, electromagnetic interfere and effect of ambient temperature [5]. Conversely, hard faults mainly happen in short- and open- circuit, or they are caused by the larger parameter variation of components [6]. The majority examples of hard faults involve the structural failure in bipolar junction transistor (BJT) and metallic oxide semiconductor field effect transistor (MOSFET) and the parameter deviation of key components in filter circuits.

Currently, there are many diagnosis approaches aiming at the two kinds of analog circuit faults. The vast majority of these methods are only implemented for field failure in factory production processes. However, the implementation of component-level diagnosis is challenging [2,6]. With respect to analog circuits, it is mainly due to the complex and changing operation conditions and external environment, such as strong electromagnetic interference, high-temperature and complicated failure mechanisms. Therefore, it is necessary to investigate an effective diagnosis method for component failure in analog electronic systems.

The rest of this paper is organized as follows. Section 2 contains a survey of the related work. In Section 3, fault feature extraction based on cross-wavelet singular entropy and parametric t-SNE is introduced. In Section 4, the algorithm and implementation procedures of the proposed PSO for parameter selection of SVM are provided. Further, fault diagnosis test in two experimental circuits is performed in Section 5 to verify the effectiveness of the proposed method. In Section 6, a discussion based on Shannon, Rényi and Tsallis entropies is presented. Finally, some conclusions are drawn in Section 7.

## 2. Related Works

Traditionally, analog circuit fault diagnoses are classified into two broad approaches: Simulation After Test (SAT) and Simulation Before Test (SBT). Compared with SAT approach, the SBT approach is more suitable for diagnostics of analog circuits as it only implements once off-line simulation process, removing on-line computation before testing and running [7]. Among SBT, data-driven diagnostic methods are based on the case that features of the system relatively changed when a fault happens. They extract features from output signals, then apply pattern recognition techniques such as neural networks (NNs) and support vector machines (SVMs) to locate a fault [8]. Meanwhile, the data-driven techniques do not need to construct an explicit model. Hence, the data-driven approaches have been applied to fault diagnosis in many relative works [9,10].

Technically, a data-driven approach can be divided into two phases: feature extraction and classifier application [11,12,13]. Obviously, feature extraction is the vital steps. To date, increasing numbers of feature extraction tools have been utilized in fault diagnosis, and they can be summarized into three categories: time-domain analysis, frequency-domain analysis, and time-frequency analysis [9,14]. Signals collected from the testing nodes of faulty circuits always carry interference components that probably overwhelm useful information. Thus, it is difficult to effectively recognize the defects of analog electronic systems when only considering the features of time-domain or frequency-domain [15]. As a typical time-frequency domain analysis, wavelet transform (WT) can reveal overlaps in time-frequency domains by decomposing the signal into a set of wavelet coefficients that vary continually over time [10]. Nevertheless, in practice, the measured signals of analog circuits commonly contain random noise, which may lead to misclassification. Therefore, it is necessary to take actions to minimize the impact of random noise. Noise removal can be executed by setting a threshold when computing wavelet coefficients [10]. However, there are some limitations: The threshold needs to be set manually, and the calculation process is time-consuming. Recently, cross-wavelet transforms (XWT) has been employed to handle partial discharge pulses and ECG signal [16,17]. Moreover, XWT has an outstanding ability in extracting time-frequency characteristics of signal and restraining noise. Consequently, XWT is applied to process the fault signals of analog circuits.

However, there are still several open issues that need to be addressed for XWT. In practical application, XWT is limited to being imported into classifiers directly because the transformed result is a high-dimension matrix. Therefore, it is necessary to combine XWT with other feature extraction techniques to reduce information abundance.

As a description of disorder or randomness of matter, entropy is capable of providing rich information about signals, which is fit for feature extraction [18,19,20]. Many scholars have devoted themselves to the field of feature extraction with use of entropy techniques. Approximative maximum entropy (Apen) has been used to diagnosis faults [9,21]. However, a bad performance could be obtained when processing the short data-set. Moreover, the Apen is sensitive to noise. Because sample entropy (Samp) is insensitive to data length and immune to noise, it can be employed as an input vector of classifiers [20,22]. However, because the Heaviside step function of sample entropy entails discontinuity at the boundary, negative results are possible. In view of this, many scholars adopt Fuzzy entropy (Fen) that vary smoothly and continuously to estimate data complexity [23]. Unfortunately, the membership function in Fen is usually difficult to determine. Some achievements in fault detection have been made using cross entropy and Rényi’s entropy [24,25], but the faulty components have not been located. Moreover, none of these techniques are used to extract features with wavelet transform. The utilization of wavelet Shannon entropy (Wse) in feature extraction is proposed, achieving a desirable performance [26]. Nevertheless, the XWT manifests a non-extensive character because of energy leakage and aliasing in the phase of wavelet operation, while Shannon entropy belongs to extensive entropy.

Based on the above, a novel feature extraction technique based on XWT and Tsallis entropy is proposed for fault diagnosis. Owning to its ability of regulating non-extensiveness, Tsallis entropy is employed to construct the feature set, denoting the complexity of fault signals [27,28]. Furthermore, to improve the efficiency of fault pattern recognition, a feasible feature reduction approach needs be implemented. A manifold learning technique is able to unearth intrinsic information embedding in highly dimensional datasets via mapping them into a low-dimensional space and retaining the local neighborhood information. Parametric t-stochastic neighbor embedding (t-SNE) has a good capability in mapping the data with high-dimension into low-dimension representation. It maintains the conditional probability distribution of data associated with the pairwise similarity from the high-dimension space to the feature subspace [29]. Therefore, it is utilized to extract discriminative features between different fault patterns.

To locate the faults, a support vector machine is employed as the classifier. SVM has advantages of high training speed and distinctive generalization ability by finding the optimal hyper-plane [30,31]. However, in practical application, it is difficult to assign the free parameter. To address this issue, various intelligent optimization algorithms, such as genetic algorithm and simulated annealing, have been utilized to determine hyper-parameters of SVM. Owing to high speed of converge and good quality of computation, quantum-behaved particle swarm optimization (QPSO) is adopted to obtain the optimal parameters [32].

## 3. Feature Extraction

### 3.1. Cross Wavelet Transform

Given a time domain signal x(t), continuous wavelet transform (CWT) can be defined as:

(1) $$W^{x}\left(a,\tau \right)=a^{-1/2}\int_{-\infty }^{+\infty }\Psi^{*}\left(\frac{t-\tau }{a}\right)dt$$

where Ψ stands for mother wavelet; * denotes complex conjugation; a(a>0) and τ are usual “dilation” and “translation” parameters.

The Morlet wavelet is a commonly used complex valued function, which can reveal the localization property of the signal in the time-frequency domain. The Morlet wavelet function can be described as follows:

(2) $$\Psi \left(t\right)=\pi^{-1/4}\left(e^{-jw_{0}t}-e^{-w_{0}^{2}/2}\right)e^{-t^{2}/2}$$

Assuming two time domain signals x(t) and y(t), the cross wavelet transform can be defined as below [33,34]

(3) $$W^{xy}\left(a,\tau \right)=W^{x}\left(a,\tau \right)W^{y*}\left(a,\tau \right)$$

Accordingly, we can plot the cross-wavelet spectrum by using the magnitude $W^{xy}\left(a,\tau \right)$ and phase $\phi =\tan^{-1}\frac{ℑ\{W^{xy}\left(a,\tau \right)\}}{ℜ\{W^{xy}\left(a,\tau \right)\}}$.

Via cross wavelet analysis, we can not only estimate the degree of correlation among signals, but also reveal the phase relationship of signals in time-frequency space.

### 3.2. Singular Value Decomposition (SVD)

On the basis of SVD theory [35], for any m×n matrix *A* can be decomposed into a m×r column-orthogonal matrix *U*, an n×r orthogonal matrix *V*, and a r×r diagonal matrix Λ, which can be described as below

(4) $$A=U\Lambda V^{T}$$

where

(5) $$\Lambda =\left[\begin{array}{ccccc} \lambda_{1} & 0 & \cdot & 0 & 0\\ 0 & \lambda_{2} & \cdot & 0 & 0\\ \cdot & \cdot & \cdot & \cdot & \cdot \\ 0 & 0 & \cdot & \lambda_{r-1} & \cdot \\ 0 & 0 & \cdot & 0 & \lambda_{r} \end{array}\right]$$

and its diagonal elements $\lambda_{i}\;\left(i=1,2,\ldots ,r\right)$ are called “singular values” of matrix *A*. The singular values are all nonnegative and arranged in a descending order (i.e., $\lambda_{1}\geq \lambda_{2}\geq \cdots \geq \lambda_{r}>0$).

### 3.3. Tsallis Entropy

For a uncertain system, the entropy is explored to estimate the uncertainty of the discrete event, which is associated with the probability distribution. Given $p=\{p_{i}\}$ denotes the probability of the system state *i*, where $0\leq p_{i}\leq 1$ and $\sum_{i=0}^{m}p_{i}=1.$ Thus, the Shannon entropy can be described as:

(6) $$S=-\sum_{i=1}^{k}p_{i}\mathrm{In}\left(p_{i}\right)$$

Besides, Shannon entropy has the extensive property:

(7) S(A+B)=S(A)+S(B)

Inspired by multi-fractal concepts, Tsallis entropy is investigate to describe non-extensive system [36], which can be expressed as

(8) $$S_{q}=\frac{1}{q-1}\left(1-\sum_{i=1}^{k}{\left(p_{i}\right)}^{q}\right)$$

where *q* stands for the entropic index, which leads to the non-extensive statistic and *k* denotes the total number of the system states.

### 3.4. Definition of XWSE

For a given time domain fault signal s(t) and template signal e(t), the detail about the feature extraction by using XWSE can be described as below:

- First, analyze the s(t) with XWT, where the “morlet” wavelet function is chosen in the process. Then, a XWT spectrum matrix *A* can be obtained by using Equations (1)∼(3).
- Second, the matrix *A* is divided into eight blocks with the same size as follows:
  $$A=\left[\begin{array}{cccc} B_{1} & B_{2} & B_{3} & B_{4}\\ B_{5} & B_{6} & B_{7} & B_{8} \end{array}\right]$$
- Third, decompose the block $B_{n}\left(n=1,2,\ldots ,8\right)$ with SVD, and a singular-value sequence for each block can be obtained as $\left\{\lambda_{1},\lambda_{2},\ldots ,\lambda_{r}\right\}$ where *r* is the rank of the diagonal matrix Λ.
- Finally, the XWSE of the block $B_{n}$ is defined by
  $$XWSE_{n}=\frac{1}{q-1}\left(1-\sum_{i=1}^{k}{\left(p_{i}\right)}^{q}\right)\;\left(n=1,2,\ldots ,8\right)$$
  where the probability $p_{i}$ associated with $\lambda_{i}$ is defined as $p_{i}=\lambda_{i}/\sum_{j=1}^{r}\lambda_{j}$. Thus, the XWSE features of fault signal s(t) can be expressed as $\left[XWSE_{1},XWSE_{2},\ldots ,XWSE_{8}\right]$

### 3.5. Parametric t-Stochastic Neighbor Embedding (Parametric t-SNE)

Given $X=\left[x_{1},x_{2},\ldots ,x_{n}\right]\in {ℜ}^{D\times n}$ is the high dimensional data set, where *D* represents the dimension of $x_{i}\;\left(i=1,2,\ldots ,n\right)$, and *n* is the number of samples. Suppose $Y=\left[y_{1},y_{2},\ldots ,y_{n}\right]\in {ℜ}^{d\times n}\;\left(d<D\right)$ denotes the low-dimensional map of *X*. By using t-SNE, the pairwise distance is transformed into the probabilities to measure the similarities between data [37,38]. In the raw space, the pairwise similarities are described as

(9) $$p_{ij}=\frac{\exp\left(-d_{H}{\left(x_{i},x_{j}\right)}^{2}\right)/2\sigma^{2}}{\sum_{k\neq l}(-d_{H}{\left(x_{k},x_{l}\right)}^{2}/2\sigma^{2}}$$

where the value of σ is determined by a binary search with a fixed perplexity. Here, the perplexity denotes the effective number of the nearest neighbors of the data $x_{i}$, and the pairwise distance $d_{H}\left(x_{i},x_{j}\right)$ represents the Euclidean distance.

In order to solve the “Crowding Problem”, the pairwise similarities are employed to described by the long-tailed student t-distribution.

(10) $$q_{ij}=\frac{{\left(1+d_{L}{\left(y_{i},y_{j}\right)}^{2}\right)}^{-1}}{\sum_{k\neq l}{\left(1+d_{L}{\left(y_{k},y_{l}\right)}^{2}\right)}^{-1}}$$

where $d_{L}\left(\cdot \right)$ stands for Euclidean distance.

Via minimizing the Kullback–Leibler divergence between two probability distributions, the cost function E(Y) is obtained to preserve the local structural characteristics of the data.

(11) $$E\left(Y\right)=\sum_{i,j}p_{ij}\log\left(p_{ij}/q_{ij}\right)$$

However, t-SNE cannot address the out-of-sample extension problem. Accordingly, the parametric t-SNE, an extension of t-SNE technique is proposed [39]. Owing to the excellent capability of the constructed nonlinear projection, Restricted Boltzmann Machines (RBMs) is adopted to construct a pre-trained parametric t-SNE network. The aim is to define a superior initialization for the fine-tuning phase. As the projection is parametric by the deep-forward network *f* with weight matrix *W*, $q_{ij}$ can be defined as follows:

(12) $$q_{ij}=\frac{{\left(1+∥f\left(x_{i}|W\right)-f\left(x_{j}|W\right){∥}^{2}/\alpha \right)}^{-\frac{\alpha +1}{2}}}{\sum_{k\neq i}{\left(1+∥f\left(x_{k}|W\right)-f\left(x_{i}|W\right){∥}^{2}/\alpha \right)}^{-\frac{\alpha +1}{2}}}$$

where α denotes the degrees of freedom of the t-distribution. Then this equation is used as the definition of $q_{ij}$ in Equation (10).

## 4. SVM and QPSO

### 4.1. Support Vector Machine (SVM)

Given a training set of N data points $\left\{\left(x_{i},y_{i}\right)\right\}$, where $x_{i}\in R^{n}$ denotes the *i*th data point, and the associated $y_{i}\in \left\{+1,-1\right\}$ represents a class label. Then, the mathematic equation of the classifier by using support vector too can be described as follows:

(13) $$y\left(x\right)=\mathrm{sign}(w^{T}\varphi \left(x\right)+b)$$

Here φ(·) stands for the kernel function which projects the input samples space into the higher dimensional feature space; *b* denotes the bias parameter, and *w* represents the weight vector of the input features.

The optimal values of *w* and *b* can be obtained by finding the solution of the following optimization problem:

(14) $$\begin{array}{c} \underset{w,b,e}{min}J\left(w,b,e\right)=\frac{1}{2}w^{T}w+\frac{C}{2}\sum_{i=1}^{N}e_{i}^{2}\\ s.t.\;y_{i}\left(w^{T}\varphi \left(x_{i}\right)+b\right)=1-e_{i},i=1,\ldots ,N \end{array}$$

where *C* denotes the regularization parameter which balance the trade-off between complexity and the proportion of non-separable samples; $e_{i}$ stands for the positive slack term for misclassification.

To address the above problem, Lagrangian function is introduced.

(15) $$L\left(w,b,e,a\right)=J\left(w,b,e\right)-\sum_{i=1}^{N}a_{i}\left\{y_{i}\left(w^{T}\varphi \left(x_{i}\right)+b\right)-1+e_{i}\right\}$$

where $a_{i}$ stands for the Lagrangian multiplier.

Finally, the decision function of the SVM classifier for any test vector $x\in R^{N}$ can be given as follows:

(16) $$y\left(x\right)=\mathrm{sign}(\sum_{i=1}^{N}a_{i}y_{i}K\left(x,x_{i}\right)+b)$$

where $K\left(x,x_{i}\right)=\varphi {\left(x_{i}\right)}^{T}\varphi \left(x\right)$ represents the kernel function. In this work, radial basis function (RBF: $K\left(x,x_{i}\right)=\exp\left(-\lambda ∥x_{i}-x{∥}^{2}\right)$) is chosen as the kernel function of the SVM classifier. Here, the term λ plays a important role on the distribution form of the samples in the high dimensional feature space.

After selecting the kernel function, the regularization parameter *C* and the RBF parameter λ should be determined. Thus, QPSO is utilized to find the optimal parameters of *C* and λ in order to improve the classification ability of SVM.

### 4.2. Quantum-Behaved Particle Swarm Optimization (QPSO)

In 1995, Ederhart and Kennedy came up with the PSO algorithm to search the optimal solutions via imitating the preying behavior of birds [40]. Nevertheless, the algorithm has some drawbacks, such as slow convergence rate and poor search ability. From the view of quantum mechanics, Sun et al. [41] have put forward QPSO. The probability of each particle’s next iteration position relies on the potential field of the particle, which is defined as below:

(17) $$X_{i}\left(t+1\right)=P\pm a|nbest-X_{i}\left(t\right)|\mathrm{In}\left(1/u\right)$$

(18) $$nbest=\frac{1}{N}\sum_{i=1}^{N}P_{i}$$

(19) $$P=sP_{i}+\left(1-s\right)P_{g}$$

where i=1,2,…,N and *N* is swarm size; *u* and *s* are uniformly distributed random numbers generated between 0 and 1; Pg is the global optimal position of all particles and Pi is the particle *i*’s optimal position; $X_{i}\left(t+1\right)$ is the position of particle *i* in iteration t+1; *nbest* is the center of all individual optimal positions; *a* is a contraction expansion coefficient.

### 4.3. The Procedure of Parameters Optimization

**Figure 1:**
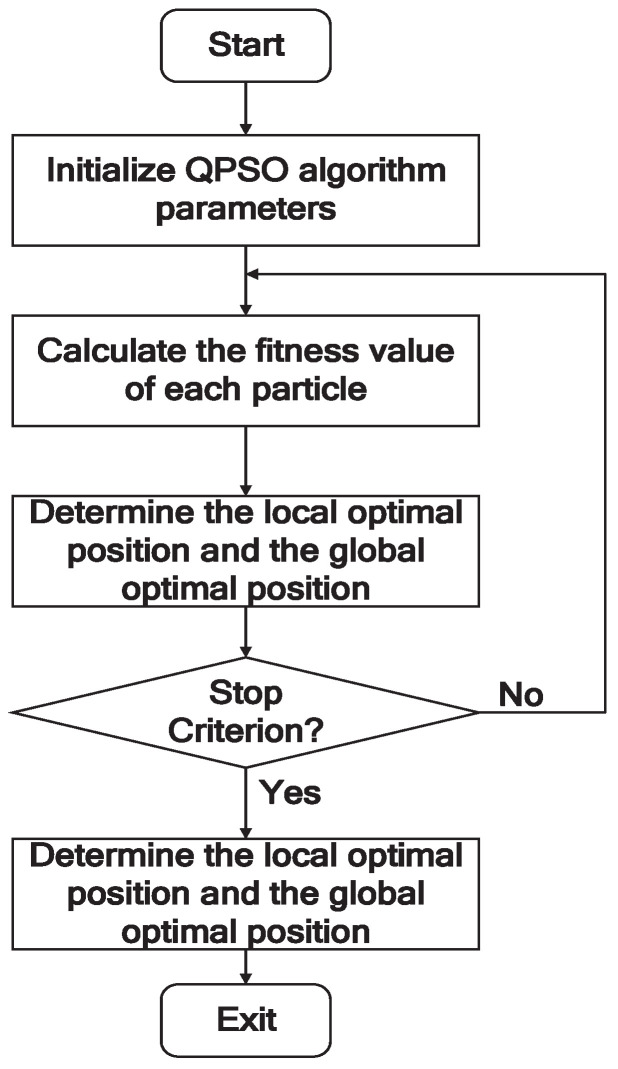
The flow chart of the parameter optimization.

This section introduces the flowchart of the QPSO algorithm-optimized support vector machine for fault diagnosis. The flowchart is shown in Figure 1 and the main steps are described as below:

**Step 1**: Initialize the QPSO algorithm parameters.

**Step 2**: For each particle, the fitness is calculated, where the cross-validation testing accuracy is used as the fitness function.

**Step 3**: Determine each particle optimal position and the global optimal position.

**Step 4**: Update the velocity and position of each particle in accordance with Equations (17)∼(19).

**Step 5**: Repeat step 2 to step 5 until reaching the stop criterion.

**Step 6**: Export the optimal 2-dimensional position as the parameters of the SVM.

**Step 7**: Exit the program.

## 5. Experimental Results and Analysis

The proposed method is investigated on three popular analog circuits in this paper. For the test circuits, each fault class is conducted 60 Monte Carlo analysis. Among these samples, 50% are used for training and the last 50% are used for testing. All testing samples are verified by an SVM classifier, then fault components can be located.

### 5.1. Example Circuits

**Figure 2:**
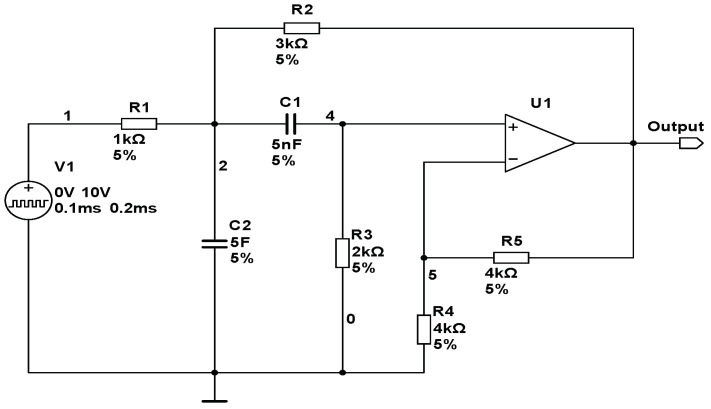
Schematic of a sallen-key band-pass filter.

**Figure 3:**
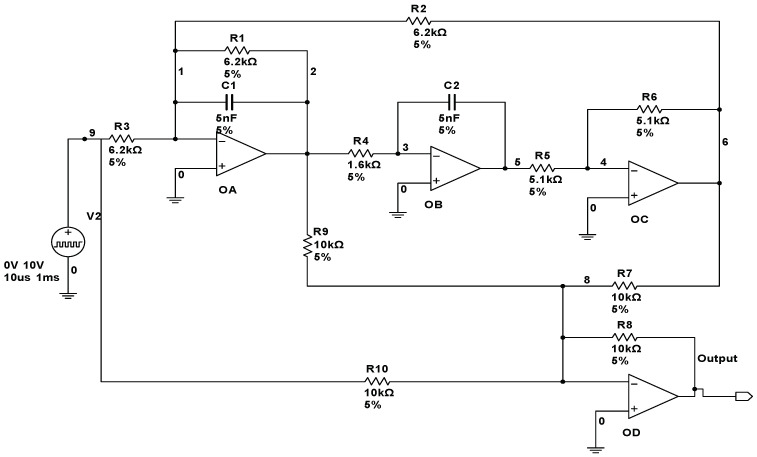
Schematic of a four-opamp filter circuit.

**Figure 4:**
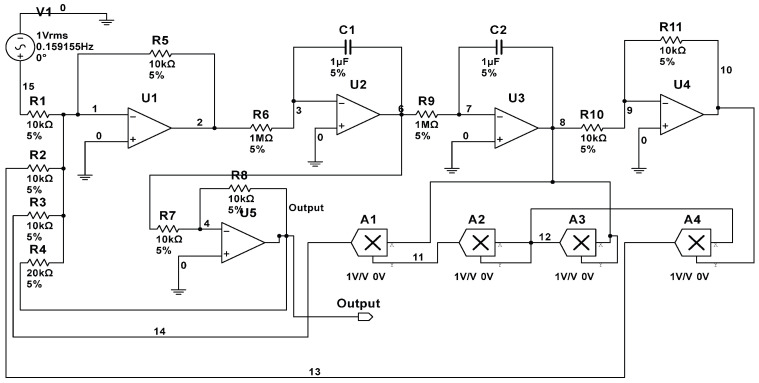
Schematic of a duffing chaotic circuit.

(1) CUT 1: The first CUT (circuit under test) shown in Figure 2 is a sallen-key band-pass circuit. In this test, the components R2, R3, C1 and C2 are chosen as fault components. The tolerances of the resistors and capacitors are all equal to 5%. A total of nine fault classes, including the fault-free (NF) status of circuits, are simulated, and the corresponding fault values and labels are shown in Table 1. In the following Table 1, Table 2 and Table 3, ↑ and ↓ refer to higher and lower than the nominal value, respectively.

(2) CUT 2: The second CUT, a four-opamps filter circuit, is shown in Figure 3. Thirteen fault classes are all shown in Table 2. The tolerances of the resistors and capacitors are also set to 5%. A pulse signal with 10 V peak, 10 µs duration and 1ms period is considered as the input signal of the circuit.

(3) CUT 3: To investigate the performance of proposed method in nonlinear circuits, a test of the duffing chaotic circuit shown in Figure 4 is conducted in this section. In this case, an excitation signal with the frequency of 0.155159 Hz and the amplitude of 0.7414148 V is chosen. The normal tolerance of resistor and capacitor is also assumed as 5%. We only collected the signals at the output node, and a 30% deviation of nominal value was considered as a fault condition. The fault modes are listed in Table 3. In this work, the test is denoted as Case 3. After data acquisition, we obtain the original samples set with size of 1080. The size of training samples set and testing samples set are all equal to 540 (30 × 18).

### 5.2. The Results Analysis of Feature Extraction

**Figure 5:**
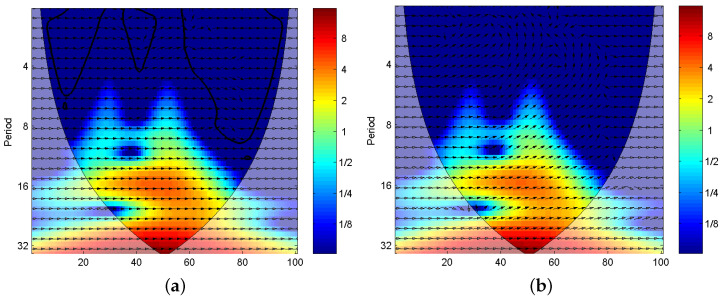
The time-frequency spectra obtained by XWT for CUT 1 (**a**) F0, (**b**) F7.

**Figure 6:**
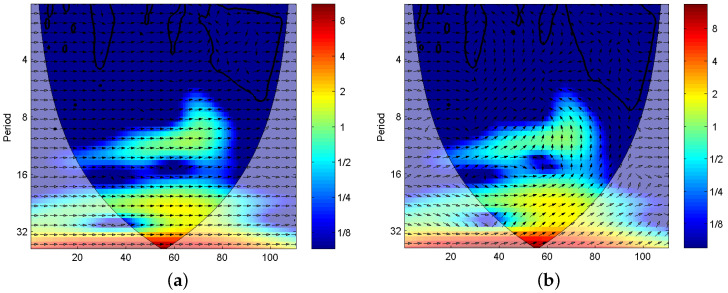
The time-frequency spectra obtained by XWT for CUT 2 (**a**) F0, (**b**) F9.

First, the sampled signals of CUTs are preprocessed by using XWT to obtain time-frequency spectra (TFS). Owing to the large quantity of fault classes, it is not feasible to list all TFS for all fault classes. Thus, we only present the TFS of F0 and F7 in Figure 5 for CUT1, and the TFS of F0 and F7 in Figure 6 for CUT2. In the figures, the color in the subgraph implies the power in the time-scale plane. And, the black arrow in each sub-image indicates the phase angle. The results from Figure 5 and Figure 6 can be concluded as follows:

(1) As shown in Figure 5, the TFSs between F0 and F7 have tiny differences. It means that the time-frequency distribution only undergos minor changes when faults happen. However, compared with the TFS of F0, the phase distributions in the TFS of F7 has an apparent difference. It indicates that the XWT can fetch phase information effectively.

(2) From Figure 6, compared with the TFS of F0, the phase distribution of F9 in the whole time-frequency plane undergoes dramatic change, and the energy accumulation block in the middle shows a considerable variation.

Consequently, with the application of cross-wavelet transform, the energy and phase characteristics in time-frequency domain can be extracted to analyze the work conditions of analog circuits.

**Figure 7:**
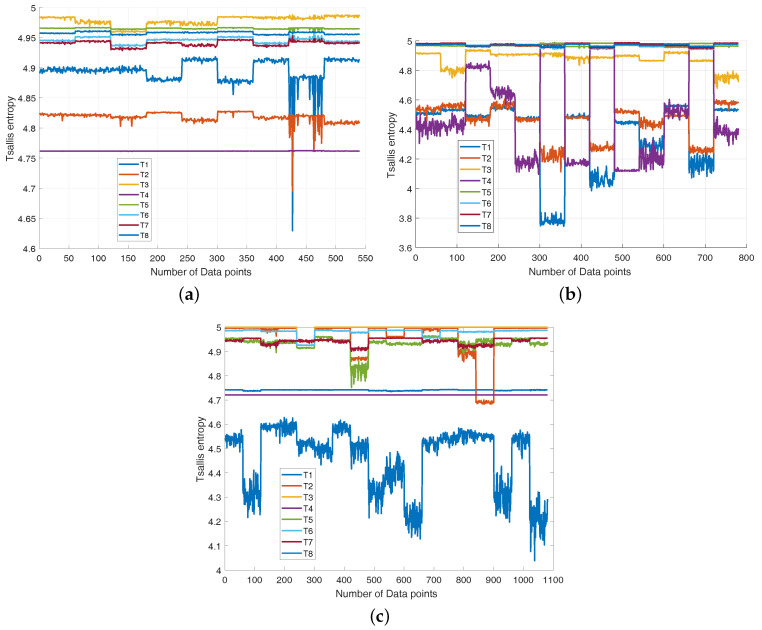
The XWSE features distribution of (**a**) CUT1, (**b**) CUT2, (**c**) CUT3.

After calculating singular entropies of blocks in the TFS, Tsallis entropy curves for CUTs are drawn in Figure 7.

As we can see from Figure 7, the eight entropies have apparent difference for all fault modes, although there exist overlapping in some points of different fault classes. It implies that Tsallis entropy can provide some discriminative information for fault recognition.

**Figure 8:**
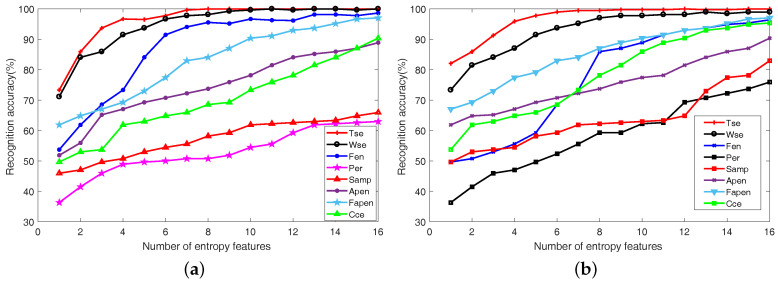
The plots of classification accuracy versus the number of features for various entropy techniques (**a**) CUT1, (**b**) CUT2.

Here, nine kinds of entropy techniques, including approximate entropy (Apen) [9], sampEn entropy (Samp) [22], fuzzy entropy (Fen) [23], permutation entropy (Per) [42], fuzzy approximate entropy (Fapen) [43], corrected conditional entropy (Cce) [43], Tsallis entropy [28] and shannon entropy [26], are employed to extract fault features, and these features are directly imported into SVM classifiers. The dimension of features is varied from 1 to 16 and finally, the resultant feature set without feature reduction are employed as the input vectors of SVM classifier. Figure 8 shows the classification rates for CUT1 and CUT 2 varying from the first features to all features. It can be observed that the recognition rate of Tsallis entropy increases steadily and achieves the highest accuracy in whole scale. Hence, it can be concluded from Figure 8 that Tsallis entropy is superior to the other entropy techniques on feature extraction.

Finally, we apply the parametric t-SNE to obtain the optimal low-dimensional representation. It not only requires less training and processing time, but also leads to a smaller structure and better generalization performance for the adopted SVM. The 220-600-600-2500-2 parametric t-SNE network structure is utilized on the fault data.

**Figure 9:**
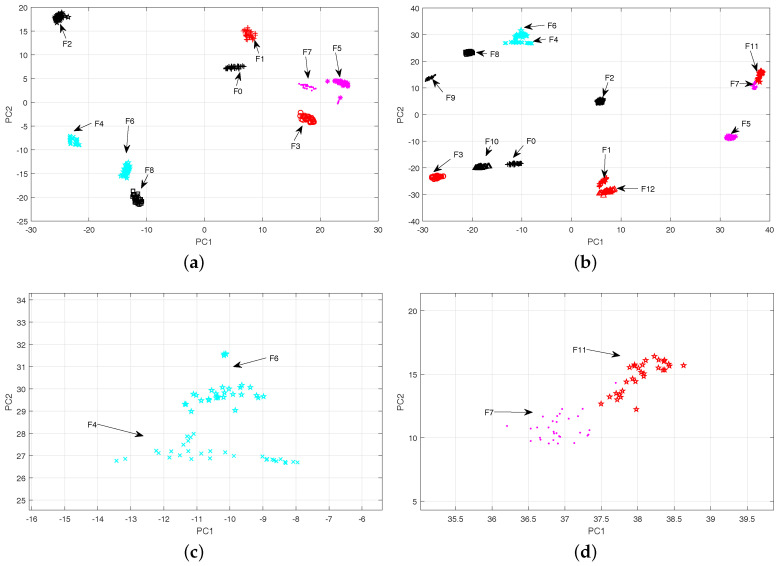
The scatter plots of two-dimensional features obtained by parametric t-SNE (**a**) CUT1, (**b**) CUT2, (**c**) F4 and F6 of CUT 2, (**d**) F7 and F11 of CUT 2.

**Figure 10:**
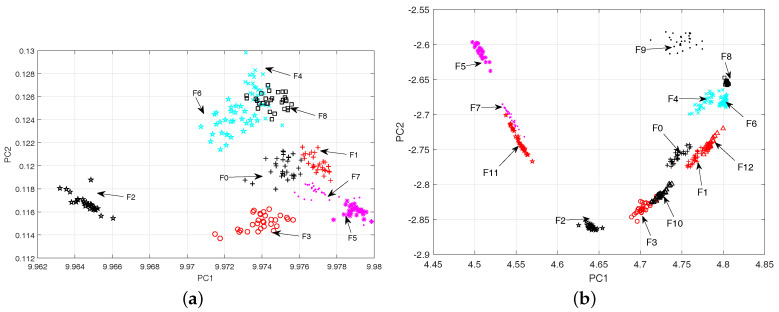
The scatter plots of two-dimensional features obtained by LPP (**a**) CUT1, (**b**) CUT2.

**Figure 11:**
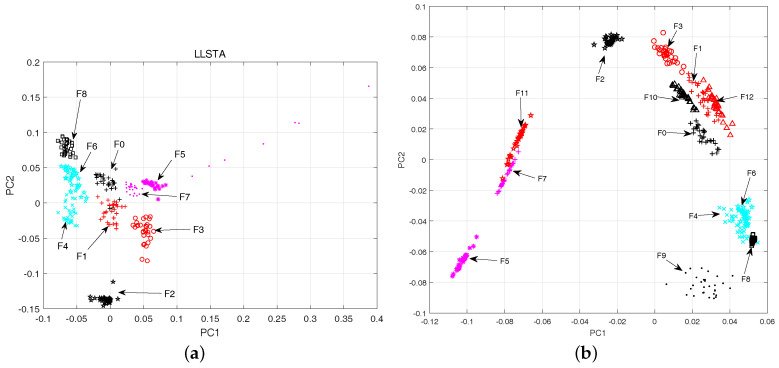
The scatter plots of two-dimensional features obtained by LLSTA(**a**) CUT1, (**b**) CUT2.

The 2-D scatter plots for the whole fault classes in CUT1 and CUT2 are shown in Figure 9. Meanwhile, the visualization of the fault data using locality preserving projection (LPP) [44] and linear local tangent space alignment (LLTSA) [45] are reported in Figure 10 and Figure 11. From Figure 9, it can be concluded that the proposed algorithm can substantially improves the separability degree of different fault classes. On the contrary, there are strong overlapping between different fault classes in Figure 10 and Figure 11. Therefore, it can be concluded that the optimal low-dimensional features can be obtained by using the Parametric t-SNE.

### 5.3. Classification Result by Using QPSO-SVM Model

**Figure 12:**
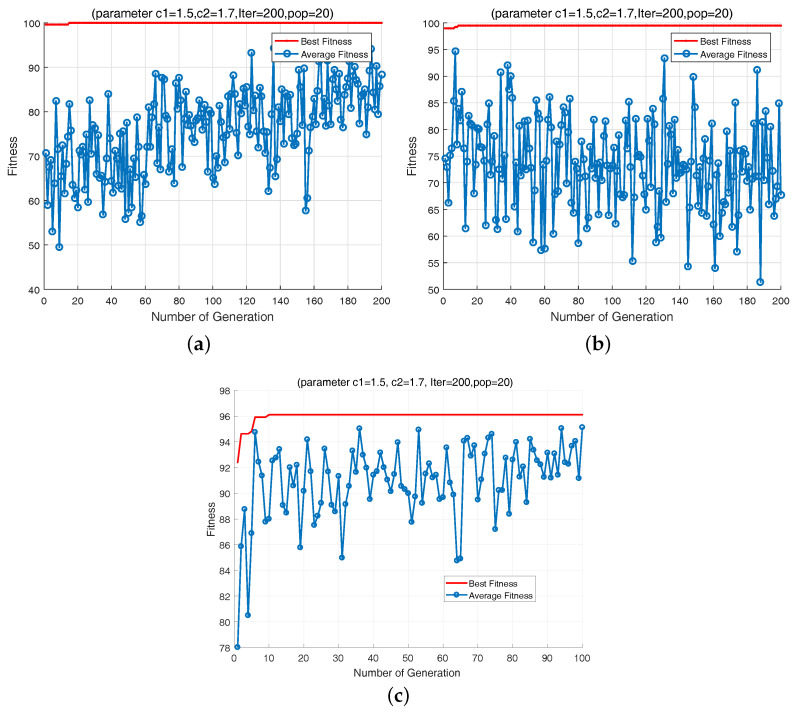
Best and the average fitness values versurs number of iterations for (**a**) CUT1, (**b**) CUT2, (**c**) CUT3.

In this study, the QPSO-based SVM is used as a classifier. After z-score normalization, the optimal features obtained by using parametric t-SNE are imported into the classifier to locate the faults. Because 60 Monte-Carlo runs are implemented for each fault class, there are 540 samples for CUT 1780 samples for CUT 2, and 1080 samples for CUT3. Each samples set is divided into two subsets with the same size. The two subsets are used as training and testing data sets, respectively. Figure 12 shows the parameter optimization procedures of these three cases. As illustrated in the figures, the presented optimization algorithm achieves desirable performances during the training stage with consuming much few time. Thus, it can be concluded that the characters in different fault classes of the circuits tend to separate obviously, and the proposed QPSO-SVM. have excellent classification ability. The optimal solutions [C,λ] for the three CUTs are [0.01, 4.076], [0.01, 86.15] and [6.5152, 0.1595] respectively.

**Figure 13:**
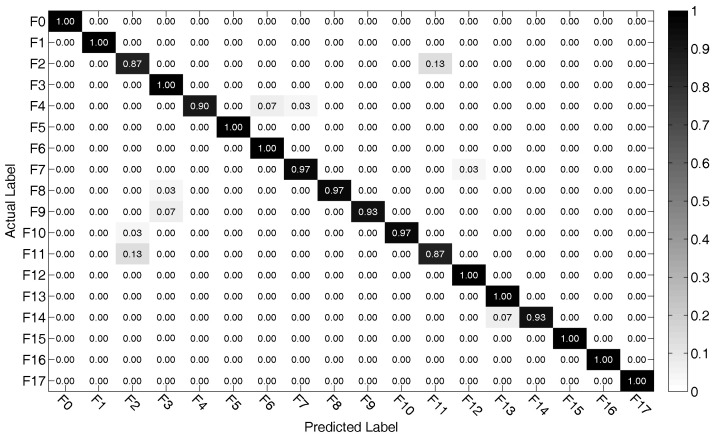
The diagnosis results of the proposed method for CUT 3.

Subsequently, the test samples are used as the input vectors of the SVM model to recognize the states. The classification accuracy comparisons with other current works for CUT1 and CUT2 are given in Table 4. Additionally, the diagnosis result of the proposed method for CUT3 is shown in Figure 13.

As shown in Table 4, it can be observed that our proposed method achieves a better result than that of other listed works, with other exceptions [4,9,10]. However, the fault components in our work have smaller parametric deviation. When fault components occur with smaller parametric deviation, the features of different fault classes tend to overlap, which results in a lower diagnosis accuracy. For the second CUT, the proposed method achieves the highest diagnostic accuracy. Therefore, with the diagnosis performance of CUT1 and CUT2, it can be summed up that the proposed scheme can effectively and accurately diagnose the soft faults in analog circuits.

As shown in Figure 13, it can be observed that some diagonal elements in the confusion matrix are close to 1. It means that the proposed algorithm has a good ability in classifying fault patterns into its actual class. However, the proposed algorithm gets unsatisfied results when dealing with some fault samples in F2 and F11. It implies that the proposed approach still needs to be improved further to fulfil the task of fault diagnosis in complex nonlinear circuits.

## 6. Discussion

Compared with Tsallis entropy, other entropy techniques, such as Rényi and Shannon entropies have already been applied to many diverse practical problems [48,49]. Therefore, a discussion based on Shannon, Rényi and Tsallis entropy is described in this section.

**Figure 14:**
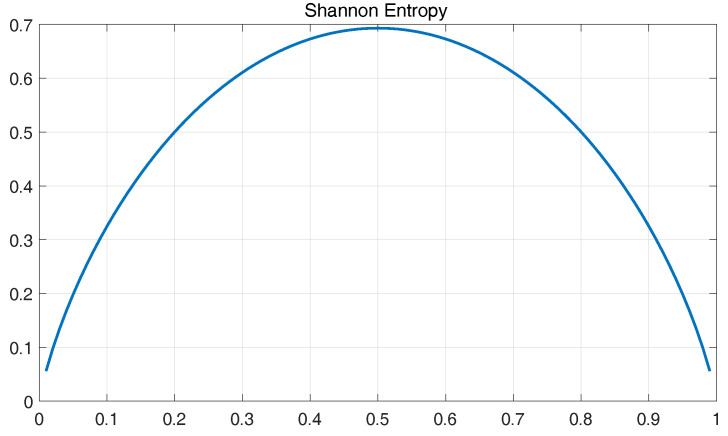
Plot of the Shannon entropy.

**Figure 15:**
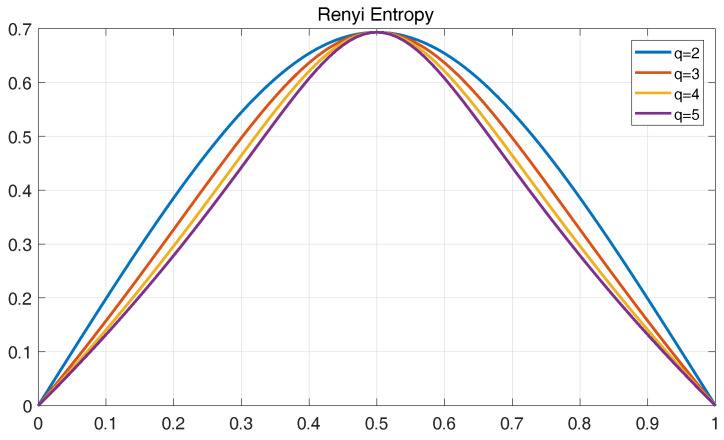
Plots of the Rényi entropy for several values of *p*.

**Figure 16:**
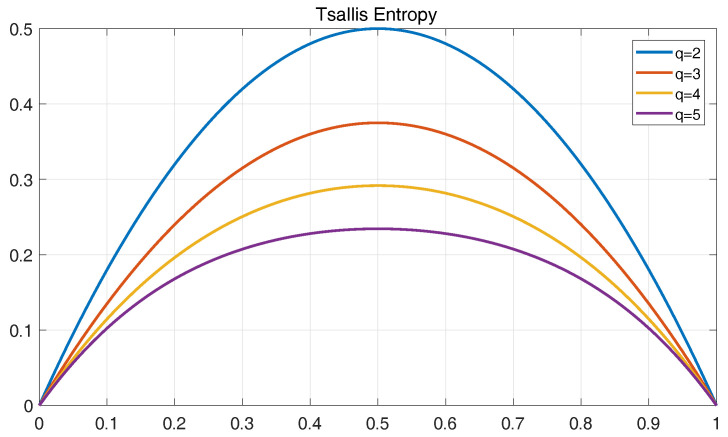
Plots of the Tsallis entropy for several values of *p*.

For given two probabilities $p_{1}$ and $p_{2}\left(p_{2}=1-p_{1}\right)$, the plots of Shannon, Rényi and Tsallis entropies are shown in Figure 14, Figure 15 and Figure 16. Here, the Rényi entropy is defined as $I_{q}=\frac{1}{1-q}\log\left(\sum_{i=1}^{n}p_{i}^{q}\right)$.

As shown in the figures, with the increase of *q*, the statistical range of Rényi entropy and Tsallis entropy will change, and the entropy values of the probability events will decrease correspondingly. However, with Shannon entropy, the statistical value of the probability events remains unchanged due to the equal weights in the entropy computation. For Tsallis entropy, the events with high probability contribute more than lower probabilities. The Rényi entropy with higher *q* parameter is determined by events with higher probabilities and the lower values of *q* coefficients weigh the events more equally.

For a signal containing noise components, the low energy components which can be used to characterize may be corrupted by the background noise that is relative to the events with small probability. In this context, Rényi and Tsallis entropies can achieve better results in extracting features by selecting appropriate *q* parameter to minimize noise as compared to Shannon entropy. Furthermore, Tsallis entropy is a much more sensitive function than Rényi entropy with respect to changes in *q* value, which is conducive to determine the proper *q* parameter. Besides, Tsallis entropy has been found to possess non-extensive property, which is helpful to deal with non-extensive character of XWT transform [50]. Based on the above advantages of the Tsallis entropy, it is applied to the fault feature extraction of analog circuits in this work.

## 7. Conclusions

In this work, a new feature extraction technique based on XWSE and parametric t-SNE is put forward, and a PSO-SVM classifier is presented to locate faults as well. The conclusions validated by the simulation experiments are drawn as blew.

- Via making full use of the time-frequency distribution characteristics and entropy description, the XWSE method has a better ability to effectively extract essential features of the analyzed fault signals, and the experimental results lead us to believe that the proposed algorithm offers great potential in revealing the difference between different fault classes.
- For the sake of eliminating useless information, the parametric t-SNE is implemented to provide a nonlinear projection from the input space to the reduced space for enhancing the feature separation degree of the fault classes. The comparisons with other dimensionality reduction methods have demonstrated its feasibility and effectiveness.
- Moreover, this work also proposes a promising means for the optimization of SVM classifier by using QPSO, which is an bionic heuristic algorithm that shows faster and better convergence rate than other methods. Simulation tests have been conducted to validate that the presented QPSO-SVM model can achieve a desirable classification performance in linear circuits as well as nonlinear circuits.

In addition to all the above achievements, several issues also need to be investigated in subsequent studies. For instance, the method of extracting features effectively under incipient and multiple faults conditions should be explored, the problem of integrating the advantages of other semi-supervised dimensionality reduction methods and parametric t-SNE needs to be studied and the performance of the proposed scheme for actual circuits fault diagnosis should be further analyzed.

## Figures and Tables

**Table 1:** Fault classes for Sallen-key bandpass filter.

Fault Code	Fault Class	Nominal Value	Faulty Value
F0	NF	-	-
F1	R2↓	3 kΩ	2.2 kΩ
F2	R2↑	3 kΩ	3.6 kΩ
F3	R3↓	2 kΩ	1.6 kΩ
F4	R3↑	2 kΩ	2.4 kΩ
F5	C1↓	5 nF	4 nF
F6	C1↑	5 nF	6.5 nF
F7	C2↓	5 nF	4 nF
F8	C2↑	5 nF	6.5 nF

**Table 2:** Fault classes for four opamp filter circuit.

Fault Code	Fault Class	Nominal Value	Faulty Value
F0	NF	-	-
F1	R1↓	6.2 kΩ	3 kΩ
F2	R1↑	6.2 kΩ	15 kΩ
F3	R2↓	6.2 kΩ	2 kΩ
F4	R2↑	6.2 kΩ	18 kΩ
F5	R3↓	6.2 kΩ	2.7 kΩ
F6	R3↑	6.2 kΩ	12 kΩ
F7	R4↓	6.2 kΩ	0.5 kΩ
F8	R4↑	6.2 kΩ	2.5 kΩ
F9	C1↓	5 nF	2.5 nF
F10	C1↑	5 nF	10 nF
F11	C2↓	5 nF	1.5 nF
F12	C2↑	5 nF	15 nF

**Table 3:** Fault classes for duffing chaotic circuit.

Fault Code	Fault Class	Fault Value
F0	-	-
F1	R1↓	7 kΩ
F2	R1↑	13 kΩ
F3	R2↓	7 kΩ
F4	R2↑	13 kΩ
F5	R3↓	7 kΩ
F6	R3↑	13 kΩ
F7	R4↓	14 kΩ
F8	R4↑	26 kΩ
F9	R8↓	7 kΩ
F10	R9↓	0.7 MΩ
F11	R10↑	13 kΩ
F12	C1↓	0.7 µF
F13	C2↑	1.3 µF
F14	R1↑R2↑	(13 kΩ) (13 kΩ)
F15	R1↓R3↓	(7 kΩ) (7 kΩ)
F16	R5↑C1↑	(7 kΩ) (1.3 µF)
F17	R6↓C2↓	(0.7 MΩ) (0.7 µF)

**Table 4:** Recongnition performance comparision of the proposed method with other existing methods.

Works	Approach	Accuracy (%)	
		CUT 1	CUT 2
Aminian et al. [1]	WT + PCA + NN	97	95
Xiao et al. [4]	FrWT + KPCA + Ridgelet−NN	100	98.52
Yuan et al. [9]	Entropy + Kurtosis + NN	100	99
Vasan et al. [10]	WT + entropy, Kurtosis + SVM	99.70	95.69
Song et al. [46]	FrFT statistical feature + SVM	98.41	95.12
Chen et al. [47]	WPT + DCQGA−SVM	97.41	98.72
Proposed	XWSE + Pt−SNE + QPSO-SVM	99.26	99.74
